# The use of etoricoxib to treat an idiopathic stabbing headache: a case report

**DOI:** 10.1186/1752-1947-1-100

**Published:** 2007-09-21

**Authors:** Mortimer B O'Connor, Elizabeth Murphy, Mark J Phelan, Michael J Regan

**Affiliations:** 1 Department of Medicine, South Infirmary – Victoria University Hospital, Old Blackrock Road, Cork, Ireland; 2 Arthritis and Osteoporosis Centre, Department of Rheumatology, South Infirmary, Victoria University Hospital, Old Blackrock Road, Cork, Ireland

## Abstract

According to the International Headache Society, idiopathic stabbing headache (ISH), an indomethacin-responsive headache syndrome, is a paroxysmal disorder of short duration manifested as head pain occurring as a single stab or a series of stabs involving the area supplied in the distribution of the first division of the trigeminal nerve. Stabs last for approximately a few seconds, occurring and recurring from once to multiple times per day in an irregular frequency, with no underlying attributable disorder.

Previously indomethacin was the principle treatment option for ISH, despite therapeutic failure in up to 35% of cases, until reports showed gabapentin, melatonin and selective cyclo-oxygenase-2 (COX-2) inhibitors were also possibly effective. In this report we present the full case report of an 88 year old lady with a history of untreated ISH where etoricoxib, a selective COX-2 inhibitor, was used to effectively treat her ISH.

## Introduction

Idiopathic stabbing headache (ISH) was first described in 1964 by Lansche [1] and over the years in has been known by many synonyms, e.g. "ophthalmodynia periodica", "ice-pick headache syndrome" and "jabs and jolts". Recently it has been included as a separate entity in the classification of the International Headache Society (IHS) – "other primary headaches". [2]

ISH is a paroxysmal disorder manifested as transient painful stabs, predominantly or exclusively felt in the distribution of the ophthalmic division of the trigeminal nerve [3]. It has a life-time prevalence in the region of 2% [4] with onset primarily in middle or late stages of life (mean age of 47 years) [5] and a female preponderance. [5]

The first line treatment of ISH has traditionally been indomethacin, which has a therapeutic failure rate in up to 35% of cases. [6] Previously novel treatments have been reported in the medical literature, including nifedipine [7], gabapentin [3], melatonin [8] and selective COX-2 inhibitors. [9] Here we present another case where the use of a selective COX-2 inhibitor was used to effectively treat ISH.

## Case presentation

An 88 year old lady presented to the Accident and Emergency department of our hospital complaining of headaches, dehydration, vomiting and general malaise for two days. Her past medical history included hypertension, Alzheimer's disease (diagnosed in 2003), right hip fracture and right wrist fracture (1996). Medications on admission included, donepezil hydrochloride 5 mg PO BD, aspirin 75 mg PO OD and trandolapril 2 mg PO OD. She had no known drug allergies. On examination she had reduced skin turgor; nil else of note. Investigations showed normal haematology, biochemistry and haematinics. Previous brain imaging was unremarkable.

On diligent history taking it was discovered the she had a history of idiopathic stabbing headache, which at the time of admission was not being treated. The nature of the history was described as follows: stabbing pains on the crown and right temporal areas, which often occurred multiple times during the day without warning and last for only a few seconds on each occasion. There were days where these headaches were not experienced. She described if the ambient temperature is elevated exacerbations may also be exacerbated, especially at night, while touching the named sites of the scalp often induces attacks of pain, making hair-care a major difficulty. There were no accompanying symptoms prior to at the time of this admission. Despite a history of Alzheimer's disease her headache history was very clear and coherent.

Due to the potential side-effect profile of indomethacin in an 88 year old patient, it was decided to commence oral etoricoxib, 60 mg given once daily with food. The patient reported a major improvement in symptoms within days. Her headache had completely resolved within a week and our records to date show no return of her symptoms.

## Discussion

Indomethacin-responsive headache syndromes represent a unique group of primary headache disorders. They can be divided into several distinct categories: a select group of trigeminal-autonomic cephalgies, valsalva-induced headaches, and ISH. The paroxysmal and continuous hemicranias invariably respond in an absolute manner to indomethacin, whereas valsalva-induced headaches and ISH may respond in an equally dramatic, but somewhat less consistent fashion. [5] The response to indomethacin is not a criterion for the diagnosis of valsalva-induced headaches and ISH, unlike in paroxysmal hemicrania where the IHS has made it an obligatory criterion. [2]

The definitive diagnosis of ISH is based upon clear details or description obtained from the history, with neuroimaging being unhelpful. The IHS criteria for diagnosis are that the pain is: (A) confined to the head, exclusively or predominantly felt in the distribution of the first division of the trigeminal nerve (orbital, temporal and parietal regions), (B) stabbing in nature and lasting fractions of a second, (C) recurring at irregular intervals (hours to days) and (D) occurring in the absence of organic disease. [2] In the region of 57% report daily episodes, 14% weekly, 23% monthly and 6% yearly episodes. [6] Episodes may last from fractions of a second (69% of cases) to rarely more than 10 seconds. [6] According to the Vaga study the majority (94.6%) last up to 3 seconds. [9] Clinically Idiopathic stabbing headache patients suffer brief, sharp, or stabbing pains that occur either as a single episode or in brief repeated volleys of pain. The pain usually lasts from 5 to 10 seconds, is of moderate severity, and may recur up to 50 times daily. [6] The syndrome may also have other manifestations such as gastrointestinal discomfort and vertigo [3], while the mechanisms in the pathologies of the syndrome remain unclear.

The current medical literature highlights that the onset of attacks of ISH is generally spontaneous but sometimes can be related to cerebrovascular disease, cranial trauma and herpes zoster. [3,9] In most sufferers the pain is unprovoked, however precipitating triggers have been recorded in the past and these include: rapid alterations in posture, physical exertion, bright light and head motion during migraine attacks. [10] This is the first reported case, known to us, where direct physical contact and a change in ambient temperature may act as triggers.

Previous studies have found that there can be a close relation between the occurrence of ISH and other headaches in adults. There is up to a 63% association with migraine [9], but also links are seen with tension-type headache (27.3%) [9] and hemicrania continua. [3] In such cases the pain is usually localized and occurs preferentially on the side most frequently affected by migraine attacks. [3]

Indomethacin has traditionally been the first line treatment for ISH, despite its unfavourable side-effects profile, and a therapeutic failure rate in at least 35% of cases [6] and partial response in 30%. [6] It is postulated that the physiology of Indomethacin possibly reduces cerebral and meningeal blood flow and antagonists effects in the nitric oxide pathways [3], and thus produces its therapeutic effect in ISH. It is recommended to start with indomethacin 12.5 mg two – three times per day, increasing the dose on alternate days until a response is apparent. [6] The majority of patients with ISH respond to a dose of indomethacin of 25 to 50 mg three times daily. [6] Recently there have been reports of other medications, with more favourable side-effect profiles, being used for ISH, namely nifedipine [7], gabapentin [3], melatonin [8] and selective COX-2 inhibitors. [9]

In this case, we did not carry out an indomethacin treatment trial due to the risks of using such a medication in the elderly [9], while etoricoxib is equipotent and better tolerated. [9]

It is difficult to be 100% certain that the clinical response seen in this case is exclusively due to etoricoxib as ISH has a natural course of spontaneous fluctuations with only 14% suffering from persistent symptoms. [6] Therefore in accordance with other COX-2 inhibitors being used effectively in ISH it may be worthwhile looking at the possibility of clinical trails in alternative treatment options. In the interim, we conclude that a selective COX-2 inhibitor, namely etoricoxib, is possibly another treatment option for ISH and may be a safer therapeutic option than indomethacin due to the lower side-effect profile. The long term safety of using a COX-2 inhibitor remains under investigation. [11]

## Competing interests

The author(s) declare that they have no competing interests.

## Authors' contributions

MOC, EM, MJR interviewed the patient. MOC, MJR prepared the paper.
